# Multienzyme One‐Pot Cascades Incorporating Methyltransferases for the Strategic Diversification of Tetrahydroisoquinoline Alkaloids

**DOI:** 10.1002/ange.202104476

**Published:** 2021-07-16

**Authors:** Fabiana Subrizi, Yu Wang, Benjamin Thair, Daniel Méndez‐Sánchez, Rebecca Roddan, Max Cárdenas‐Fernández, Jutta Siegrist, Michael Richter, Jennifer N. Andexer, John M. Ward, Helen C. Hailes

**Affiliations:** ^1^ Department of Chemistry University College London 20 Gordon Street London WC1H 0AJ UK; ^2^ Department of Biochemical Engineering University College London Bernard Katz Building London WC1E 6BT UK; ^3^ Institute of Pharmaceutical Sciences University of Freiburg Albertstr. 25 79104 Freiburg Germany; ^4^ Fraunhofer Institute for Interfacial Engineering and Biotechnology (IGB) Branch Biocat Schulgasse 11a 94315 Straubing Germany

**Keywords:** alkaloids, biocatalysis, methyltransferases, one-pot cascades, regioselectivity

## Abstract

The tetrahydroisoquinoline (THIQ) ring system is present in a large variety of structurally diverse natural products exhibiting a wide range of biological activities. Routes to mimic the biosynthetic pathways to such alkaloids, by building cascade reactions in vitro, represents a successful strategy and can offer better stereoselectivities than traditional synthetic methods. *S*‐Adenosylmethionine (SAM)‐dependent methyltransferases are crucial in the biosynthesis and diversification of THIQs; however, their application is often limited in vitro by the high cost of SAM and low substrate scope. In this study, we describe the use of methyltransferases in vitro in multi‐enzyme cascades, including for the generation of SAM in situ. Up to seven enzymes were used for the regioselective diversification of natural and non‐natural THIQs on an enzymatic preparative scale. Regioselectivites of the methyltransferases were dependent on the group at C‐1 and presence of fluorine in the THIQs. An interesting dual activity was also discovered for the catechol methyltransferases used, which were found to be able to regioselectively methylate two different catechols in a single molecule.

## Graphical abstract

In vitro multi‐enzyme cascade reactions were developed leading to the scalable synthesis of methylated tetra‐hydroisoquinoline alkaloids. The reactions occurred in one‐pot starting from simple building blocks such as dopamine or l‐tyrosine. The use of selected methyltransferases enabled regioselective methylations. When more than one catechol moiety was present, methylations could be directed towards one or more hydroxyl group.

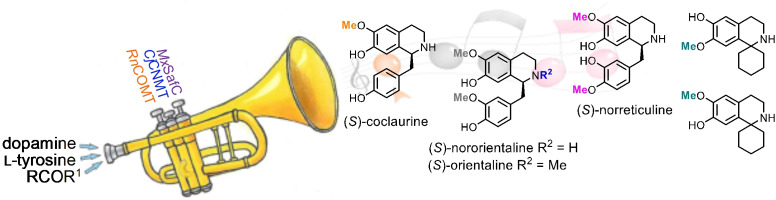

## Introduction

THIQs are an important group of bioactive alkaloids. Alongside their applications as analgesics and antitussives,[^1^, ^2^, ^3^, ^4^] THIQs show promise as therapeutics towards cancer, neuropathologies and multi‐drug resistant bacteria.[^3^, ^5^, ^6^, ^7^, ^8^] However, supply of these compounds for studies and clinical use is limited. Natural THIQs can be harvested from mixtures from certain plants, but their chemical syntheses are complicated by the presence of chiral centres and the density of functional groups.^[9]^ An enzyme of particular interest has been norcoclaurine synthase (NCS), which catalyses a Pictet–Spengler (PS) reaction between dopamine and 4‐hydroxyphenylacetaldehdye, to initiate benzylisoquinoline alkaloid (BIA) biosynthesis. Most usefully, using natural and engineered NCS variants,[^10^, ^11^, ^12^] the β‐arylethylamine and carbonyl substrates can be varied to generate a number of natural and non‐natural THIQs.[^13^, ^14^, ^15^, ^16^] The diversity of THIQs generated has been further extended by forming in vitro cascades with enzymes upstream of NCS.^[17]^ In plant BIA biosynthesis, methyltransferase (MT) enzymes operate downstream of NCS to methylate norcoclaurine.^[18]^ Such natural product methylations involve transfer of a methyl group from *S*‐adenosylmethionine (SAM) to specific nucleophilic sites on the scaffold, and is widely associated with activation of the compound or improvement of its biological properties.[^19^, ^20^, ^21^, ^22^, ^23^, ^24^]

**Figure 1:**
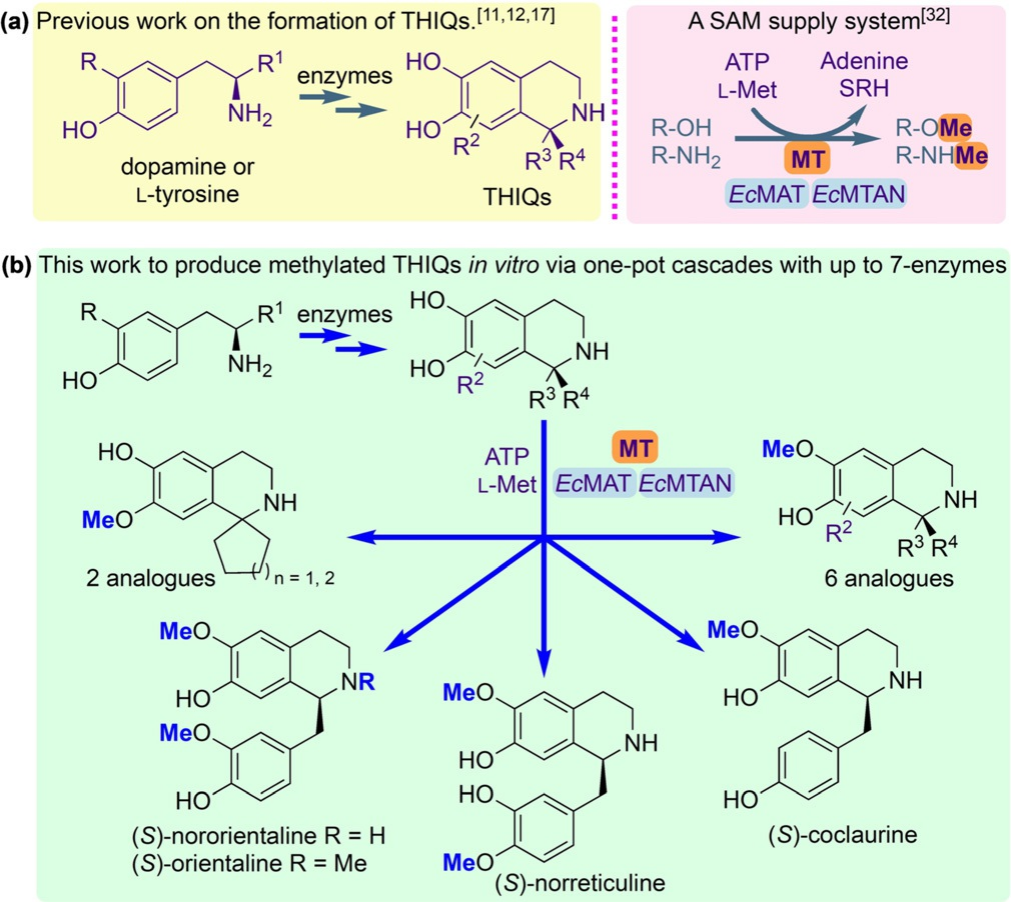
(a) Formation of THIQs using enzymes and *O*‐ or *N*‐methylation using methyltransferases (MTs) and an *S*‐adenosylmethionine (SAM) supply/*S*‐adenosylhomocysteine (SAH) degradation system; (b) The route to biologically relevant methylated THIQ*s* prepared in this work via cascades with up to 7‐enzymes. Abbreviations: *E. coli* (*Ec*); methionine adenosyltransferase (MAT); methylthioadenosine nucleosidase (MTAN); L‐methionine (L‐Met); adenosine triphosphate (ATP); *S*‐ribosyl‐L‐homocysteine (SRH).

MTs are therefore an essential addition for the diversification of THIQs. However, while the *O*‐MTs involved in producing natural BIAs are regioselective,[^25^, ^26^] they lack the substrate scope in vivo needed to be components of a flexible enzyme cascade to larger sets of THIQs. While in vitro biocatalytic cascades take inspiration directly from natural biosynthetic pathways, they allow more flexibility over reaction design, offering control in the order and quantity of enzymes added to the cascade as well as the enzyme used. Importantly, the in vitro approach reduces background reactions such as the phosphate catalysed PS,^[27]^ and can simplify product isolation and purification. Here we present the use of three *O*‐MTs in vitro, catechol‐*O*‐MTs from *Rattus norvegicus* (*Rn*COMT),^[28]^ *Coptis japonica* (*Cj‐*6‐OMT),[^25^, ^29^] and *Myxococcus xanthus* (*Mx*SafC),^[30]^ and an *N*‐MT from *Coptis japonica* (*Cj*CNMT),^[31]^ to establish potential reactivities towards a range of THIQs. We report the integration of these enzymes into up to 7‐step cascades for the strategic diversification of bioactive compounds (Figure 1). The cost of SAM has also previously limited the scale of MT‐catalysed reactions, but this challenge is resolved with the incorporation of a reported modular in vitro cofactor supply system,^[32]^ demonstrating the capacity of such an approach in ambitious biocatalytic applications.

## Results and Discussion

### Initial *O*‐MT Screening

**Figure 2:**
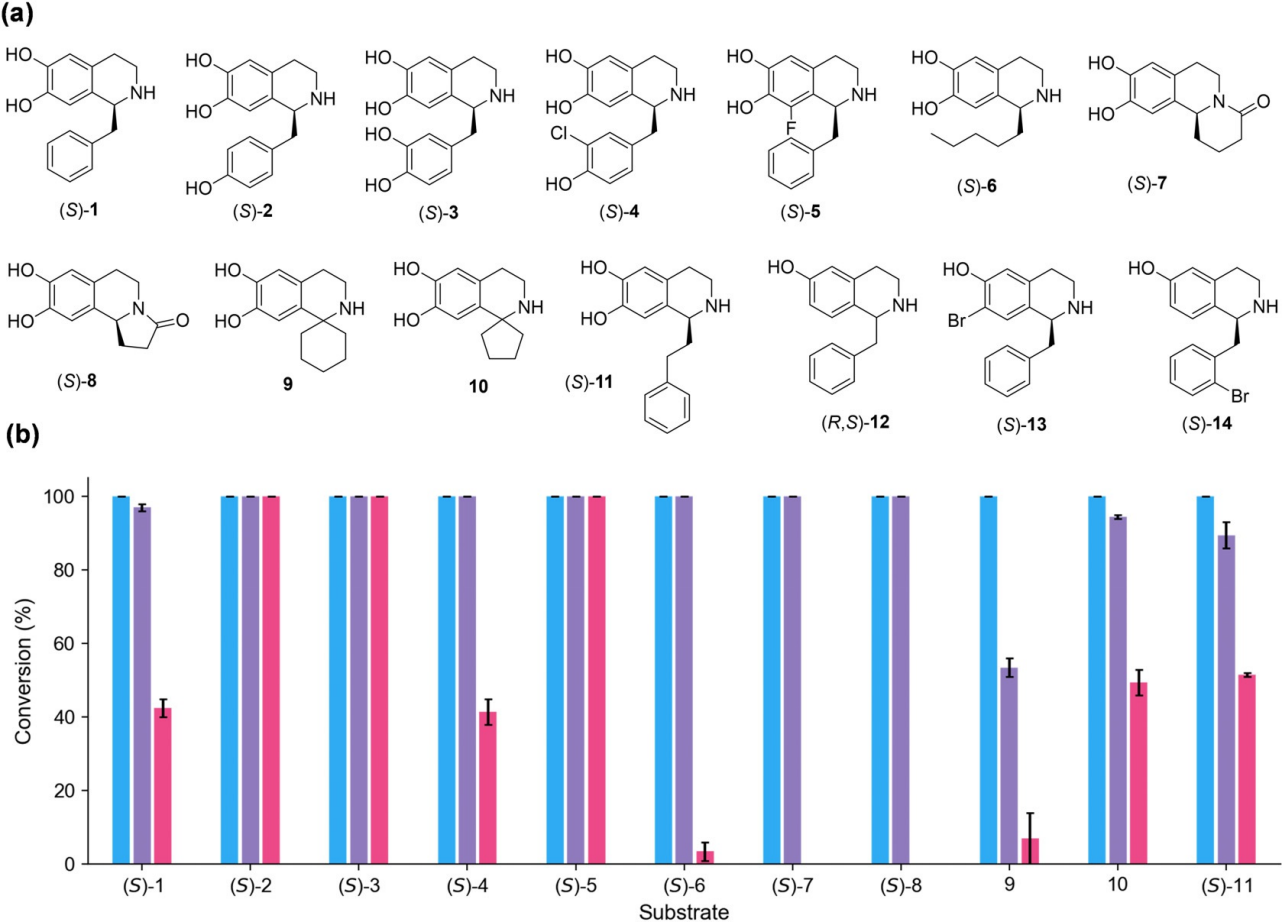
Substrates used with the selected MTs indicated. (a) Structures of the THIQs tested. (b) Reaction conversions are based on the consumption of the THIQ analogues **1**–**14** used, by HPLC analysis against standard calibration curves. Reaction conditions: THIQ 0.5 mM, SAM 3 mM, and selected MTs (clarified lysate, 10 % v/v) which showed conversions after 90 min: *Rn*COMT in blue, *Mx*SafC in purple, and *Cj*‐6‐OMT in pink. THIQs (*R*,*S*)‐**12**, (*S*)‐**13**, and (*S*)‐**14** were not accepted by any of the MTs tested and are not reported. All reactions were performed in duplicate. See SI for representative HPLC chromatograms and calibration curves.

In the initial screening, a variety of THIQs were prepared using *Thalictrum flavum* NCS (*Tf*NCS), in high enantiomeric excesses (*ee*s) where the single isomer is indicated, and purified.[^11^, ^16^, ^17^] The substrate panel (Figure 2 a) included BIAs (**1**–**5**) and C‐1 substituted THIQs with linear aliphatic or cyclic moieties (**6**–**11**). BIAs lacking the catechol group on the isoquinoline scaffolds or those bearing a halide group (**12**–**14**) were also included. All substrates **1**–**14** were tested with MT enzymes *Rn*COMT and *Mx*SafC. *Cj‐*6‐OMT was also used for comparison as it is known to selectively methylate in vivo the 6‐OH of norcoclaurine (*S*)‐**2**.^[29]^ Initially, purified enzymes were used for reactions with substrates **1**–**3**. With the aim of scaling up the process and making it more viable in an industrial setting, the same reactions were also carried out with clarified lysates, showing regioselectivities that were comparable to the purified *O*‐MTs (SI section 3.2). Clarified lysates were then used with all the substrates. We were delighted to see that all THIQs bearing a catechol were readily accepted and new peaks were detected by HPLC within the first hour (Figure 2 b and SI Figure S5). In general, *Rn*COMT and *Mx*SafC showed broader substrate scope relative to *Cj‐*6‐OMT, with excellent activities towards THIQs with an aliphatic side chain (**6**–**10**), which were only slightly or not converted by *Cj‐*6‐OMT under these conditions. Notably, complete conversions were obtained within the first 90 min with substrates **1**–**11** with *Rn*COMT, while *Mx*SafC showed some starting material left only in the case of substrates **9**–**11**. As expected, none of the THIQs lacking a catechol moiety (**12** and **14**) were accepted by the MTs, including derivative **13** where a catecholic hydroxyl group was replaced with a bromine.

### Building the Enzyme Cascades and *O*‐MT Regioselectivities

**Scheme 1:**
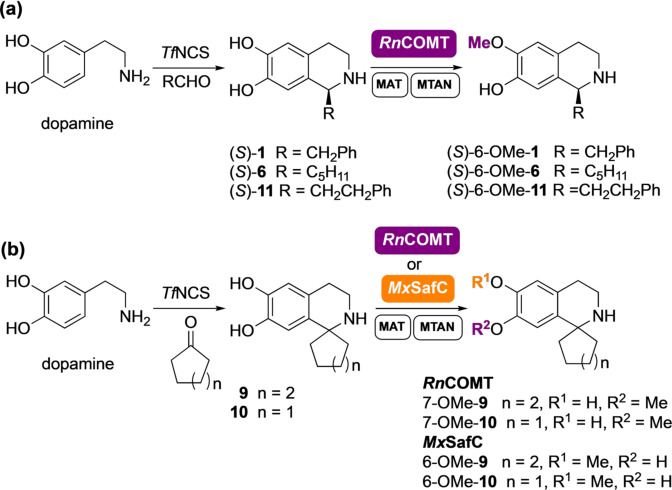
Four‐enzyme cascades for the synthesis of: (a) THIQs (*S*)‐6‐OMe‐**1**, (*S*)‐6‐OMe‐**6**, and (*S*)‐6‐OMe‐**11**, and (b) THIQs 6‐OMe‐**9**, 7‐OMe‐**9**, 6‐OMe‐**10**, and 7‐OMe‐**10** from **9** and **10** respectively.

Establishment of the regiochemistries was also key, which required scale‐up of the reactions for product characterisation. Several factors were considered. Firstly, the high cost of the cofactor SAM restricts its use in larger scale biocatalytic reactions in vitro: although it has been reported in the *N*‐methylation of THIQs,^[31]^ this is not practicable for wider applications. In previous work, integration of MTs into a linear cascade with two further enzymes overcame this issue using a methionine adenosyltransferase (MAT E.C. 2.5.1.6) and a methylthioadenosine nucleosidase (MTAN, E.C. 3.2.2.9).^[33]^ MATs generate SAM from ATP and L‐methionine, both of which are less expensive and importantly more stable than SAM. When SAM is used for the methylation reaction, *S*‐adenosylhomocysteine (SAH) is formed as a by‐product. SAH, which would otherwise inhibit the methylation, can then be cleaved by the MTAN into *S*‐ribosyl‐L‐homocysteine (SRH) and adenine (Figure 1 a). For the SAM supply/SAH degradation system, enzymes MAT and MTAN, both from *E. coli* (*Ec*MAT and *Ec*MTAN, respectively) were selected.^[33]^ Secondly, clarified lysates were used instead of pure enzymes to avoid the requirement for enzyme purification steps. Thirdly, to further streamline the process, the methylation cascade reaction was coupled directly to the NCS‐catalysed PS reaction in a one‐pot, two‐step cascade, obviating the need for purification of the THIQ intermediates. The methylation of representative substrates (*S*)‐**1**, (*S*)‐**6**, **9**, **10** and (*S*)‐**11** was explored first (Scheme 1. *ee*s for (*S*)‐**1** (>97 %) and (*S*)‐**6** (>98 %) are shown in the SI). Here it was essential that all the dopamine was consumed in the *Tf*NCS catalysed reaction before the MT step to avoid the methylation of unreacted dopamine,^[33]^ which could interfere with the regiochemical outcome.

With the architecture of the cascades defined, a biocatalytic scale‐up of the methylated THIQs was attempted in a single pot. Starting from dopamine (5–20 mM) and an excess of the corresponding carbonyl compound, the THIQs (*S*)‐**1**, (*S*)‐**6**, **9**,**10** and (*S*)‐**11** were generated with high enantiopurity (for single isomer compounds) and the methylation step initiated by the addition of *Rn*COMT clarified lysate (10 % v/v) in the presence of ATP, L‐methionine and the SAM supply/SAH degradation system enzymes *Ec*MAT (10 % v/v) and *Ec*MTAN (2.5 % v/v). Using *Rn*COMT the methylated products (*S*)‐6‐OMe‐**1**, (*S*)‐6‐OMe‐**6**, 7‐OMe‐**9**, 7‐OMe‐**10**, and (*S*)‐6‐OMe‐**11** were isolated with good yields and high regioselectivities with only 5–10 % of the opposite regioisomer detected by ^1^H NMR spectroscopy in most cases (Table 1). As expected, the regiochemistry of the reaction was strongly dependent on the nature of the side chain, which directed the methylation preferentially at the 6‐OH for substrates (*S*)‐**1**, (*S*)‐**6**, and (*S*)‐**11,** and surprisingly preferably on the 7‐OH for substrates **9** and **10**. Interestingly, when **9** or **10** underwent the same reaction conditions with *Mx*SafC, the 6‐OH was methylated instead preferentially and derivatives 6‐OMe‐**9** or 6‐OMe‐**10** respectively were obtained in good yield and excellent regioselectivities (Table 1). This unique performance with a switch in the regioselectivities for both *Rn*COMT and *Mx*SafC further confirms the complementarity of these two enzymes.^[33]^

**Table 1:** Isolated yields and regioisomeric ratios for the preparative scale cascade reactions described in Schemes 1–3.

Substrate	Product	MT	Yield^[a]^	Regioisomeric ratio^[b]^ (6‐OMe:7‐OMe)
(*S*)‐**1**	(*S*)‐6‐OMe‐**1**	*Rn*COMT	55 % (96 %)	95:5
(*S*)‐**2**	(*S*)‐6‐OMe‐**2**	*Rn*COMT	55 % (92 %)	95:5
(*S*)‐**2**	(*S*)‐6‐OMe‐**2**	*Mx*SafC^[c]^	43 % (89 %)	60:40
(*S*)‐**2**	(*S*)‐6,7‐(OMe)_2_‐**2**	*Mx*SafC^[c]^	6 % (9 %)^[d]^	na
(*S*)‐**3**	(*S*)‐6,3′‐(OMe)_2_‐**3**	*Rn*COMT	34 % (64 %)^[e]^	95:5
(*S*)‐**3**	(*S*)‐6,4′‐(OMe)_2_‐**3**	*Cj‐*6‐OMT then *Mx*SafC	27 % (70 %)^[e]^	100
(*S*)‐**5**	(*S*)‐6‐OMe‐**5**	*Rn*COMT	19 % (27 %)	95:5
(*S*)‐**5**	(*S*)‐6‐OMe‐**5**	*Mx*SafC	20 % (31 %)	95:5
(*S*)‐**6**	(*S*)‐6‐OMe‐**6**	*Rn*COMT	44 % (90 %)	90:10
**9**	7‐OMe‐**9**	*Rn*COMT	40 %	10:90
**9**	6‐OMe‐**9**	*Mx*SafC	56 % (94 %)^[f]^	90:10
**10**	7‐OMe‐**10**	*Rn*COMT	45 %^[g]^ (90 %)	10:90
**10**	6‐OMe‐**10**	*Mx*SafC	30 %^[g]^ (89 %)	90:10
(*S*)‐**11**	(*S*)‐6‐OMe‐**11**	*Rn*COMT	33 % (40 %)	85:15

[a] Isolated yield (yield by HPLC analysis against product standards in parenthesis); [b] 6‐OMe:7‐OMe calculated by NMR; [c] reaction with 2 equivalents of ATP and L‐methionine; when an excess of methyl equivalents was used the dimethylated product (*S*)‐6,7‐(OMe)_2_‐**2** was observed; [d] the yield of (*S*)‐6,7‐(OMe)_2_‐**2** with 8 equiv of ATP and L‐methionine was 28 % (47 %); [e] some impurities were detected by HPLC; [f] reaction on a pure sample of **9**. When carried out in a cascade coupled with NCS the yield was 64 % (not optimised); [g] reaction on a pure sample of **10**.

**Scheme 2:**
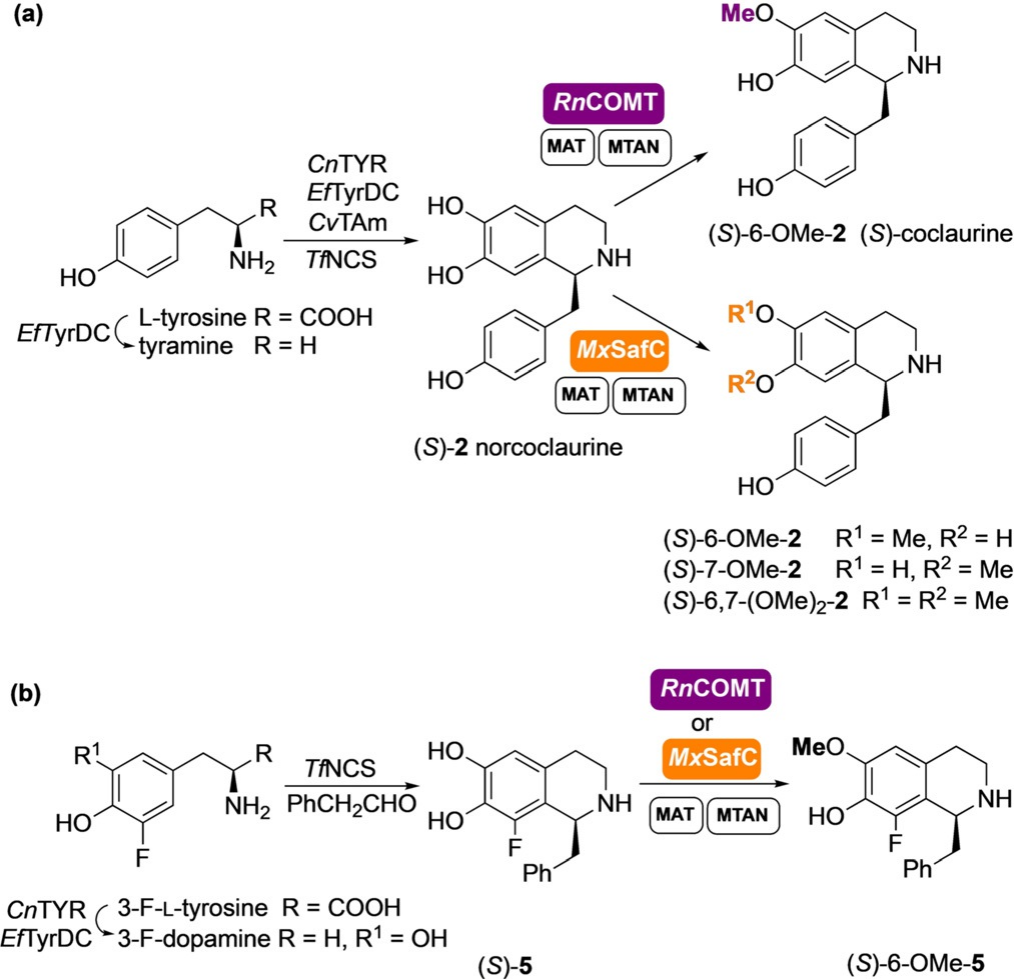
(a) Seven‐enzyme cascades for the synthesis of THIQs (*S*)‐6‐OMe‐**2**, (*S*)‐7‐OMe‐**2** and (*S*)‐6,7‐(OMe)_2_‐**2** and (b) six‐enzyme cascade for the synthesis of THIQs (*S*)‐6‐OMe‐**5** on a laboratory preparative scale.

With the challenge to demonstrate the potential of this approach, the methylation with *Rn*COMT was further coupled with a multistep in vitro enzyme cascade for the in situ generation of norcoclaurine (*S*)‐**2** (generated in >97 % *ee*) using *Candidatus* Nitrosopumilus salaria BD31 tyrosinase (*Cn*TYR) and *Enterococcus faecalis* DC32 tyrosine decarboxylase (*Ef*TyrDC), together with a versatile transaminase from *Chromobacterium violaceum* ^[34]^ (*Cv*TAm) and wild‐type *Tf*NCS enzyme.^[17]^ A total of seven enzymes as clarified cell‐lysates were used in the same pot, achieving the conversion of L‐tyrosine into (*S*)‐coclaurine (*S*)‐6‐OMe‐**2** in an unique one‐pot three step cascade (Scheme 2 a).

The methylation with *Rn*COMT was almost completely regioselective with no appreciable regioisomeric peak detected by HPLC. When the cascade was scaled up to 50 mL (L‐tyrosine 20 mM), (*S*)‐6‐OMe‐**2** was isolated in 55 % yield and small amounts (5 % of total product) of the 7‐*O*‐methylated regioisomer were detected by ^1^H NMR spectroscopy. The lower isolated yields compared to the complete conversion of the starting material and the almost quantitative yield measured by HPLC, reflects again the challenge of purifying such alkaloids.^[35]^ Catechol *O*‐MTs such as *Rn*COMT have not been shown to methylate adjacent hydroxy groups or phenols, even in the presence of an excess of methyl equivalents. Interestingly, the structurally related *Mx*SafC, proved to be an exception. This consideration derives from the fact that methylation of (*S*)‐**2** with *Mx*SafC and an excess of SAM, led to a regioisomeric mixture of (*S*)‐6‐OMe‐**2** and (*S*)‐7‐OMe‐**2** (ratio 60:40), which were converted over time into a third product. This unexpected product was isolated and characterized confirming the structure of the 6,7‐dimethoxy tetrahydroisoquinoline (*S*)‐6,7‐(OMe)_2_‐**2** (see the SI).

A one‐pot cascade was also established for the synthesis and methylation of (*S*)‐**5** (formed in 90 % *ee*) (Scheme 2 b). *Cn*TYR and *Ef*TyrDC were used for the hydroxylation and decarboxylation of 3‐F‐L‐tyrosine,^[17]^ then reaction with phenylacetaldehyde and *Tf*NCS followed by methylation with *Rn*COMT on the crude reaction mixture, gave the 6‐*O*‐methylated derivative (*S*)‐6‐OMe‐**5** which was isolated in 19 % yield (27 % HPLC yield) over the 7‐enzyme steps. Similarly, using *Mx*SafC gave (*S*)‐6‐OMe‐**5** in 20 % yield (31 % HPLC yield). The limiting step is most likely the initial hydroxylation with *Cn*TYR. The preferential methylation at the 6‐OH position by both MTs is likely a consequence of substrate‐active site interactions. However, the change in *Mx*SafC regioselectivity between (*S*)‐**1** and fluorinated (*S*)‐**5** indicates electronic factors are also important. The fluorine adjacent to a catechol moiety decreases the p*K*_a_ of the neighbouring ‐OH and has previously been shown to direct the methylation on the hydroxyl group to a position equivalent to that at 7‐OH.^[36]^ Here, the fluorine at C‐8 in the THIQ scaffold directs the methylation unexpectedly towards (*S*)‐6‐OMe‐**5** with *Mx*SafC. Notably, *Mx*SafC led to a mixture of the two regioisomeric products when (*S*)‐**1**, lacking the fluorine, was used as starting material. We hypothesized that a subtle substrate conformational change together with an intramolecular hydrogen bonding between the fluorine and the adjacent ‐OH, can make methylation at that position more difficult.

### Extending the Enzyme Cascades for Further *O‐ and N*‐methylations

**Scheme 3:**
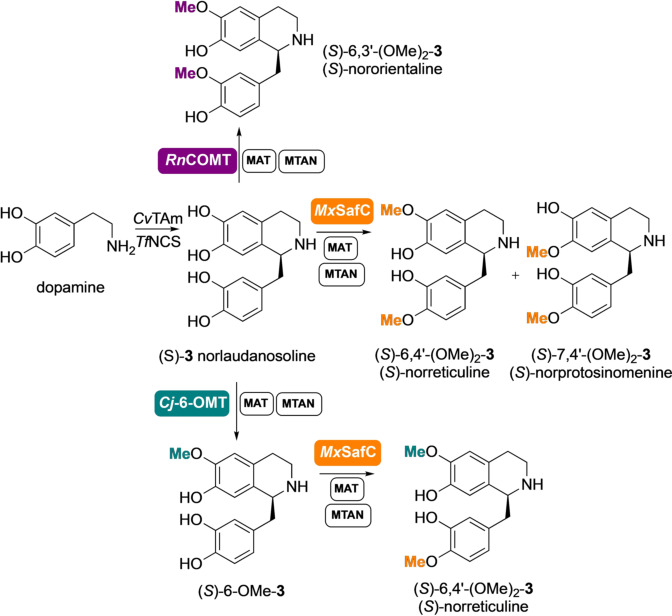
Enzymes cascades for the synthesis of THIQs (*S*)‐6,3′‐(OMe)_2_‐**3**, (*S*)‐6,4′‐(OMe)_2_‐**3**, and (*S*)‐7,4′‐(OMe)_2_‐**3** on a laboratory preparative scale.

A case of particular interest is norlaudanosoline (*S*)‐**3**. This is a substrate for *Cj‐*6‐OMT and it is converted to the corresponding 6‐*O*‐methylated derivative (*S*)‐6‐OMe‐**3**.^[29]^ When the assay was performed with either *Rn*COMT or *Mx*SafC and an excess of SAM, new product peaks not attributable to the 6‐ or 7‐*O*‐methylated products were identified by HPLC. Interestingly, when only one equivalent of SAM was used with *Rn*COMT, the peak corresponding to 6‐*O*‐methylated **3** was identified as the main product together with some starting material. If *Mx*SafC was used instead, a mixture of two regioisomeric methylated products was identified (SI Figure S5). With the aim of elucidating the structure of the new products, a two‐step, five‐enzyme cascade was established for the synthesis and methylation of norlaudanosoline (*S*)‐**3** (formed in >97 % *ee*) in one pot (Scheme 3) starting from dopamine (20 mM). The wild‐type *Tf*NCS enzyme and *Cv*TAm^[34]^ were used in the first step,^[37]^ and the methylation step was then coupled and scaled up using *Rn*COMT together with the SAM supply/SAH degradation system. Product isolation confirmed that a second methylation occurred on the catechol moiety of the C‐1 side chain and *Rn*COMT proved to be exceptionally selective, with methylation preferentially occurring at the 6‐OH and the 3′‐OH. Complete conversion into nororientaline (*S*)‐6,3′‐(OMe)_2_‐**3** was observed, which was isolated in 33 % yield. This double methylation is completely unprecedented as it is known to be carried out by two different MTs in the BIA biosynthetic pathway, with *Rn*COMT combining the activity of a 6‐OMT and a 3′‐OMT.^[38]^ *Mx*SafC showed a similar performance, leading to a mixture of 6‐OMe and 7‐OMe‐(*S*)‐norlaudanosoline which were then converted into the corresponding dimethylated analogues (*S*)‐norreticuline (*S*)‐6,4′‐(OMe)_2_‐**3** and (*S*)‐norprotosinomenine^[39]^ (*S*)‐7,4′‐(OMe)_2_‐**3** in the presence of an excess of methyl equivalents.^1^ Although less selective, *Mx*SafC could also combine the activity of two methyltransferases, including a 4′‐OMT. This reactivity was used for the selective synthesis of (*S*)‐norreticuline (*S*)‐6,4′‐(OMe)_2_‐**3** by coupling the *Cj‐*6‐OMT and the *Mx*SafC in a one‐pot three‐step cascade (Scheme 3), highlighting the ability to mix and match MTs to directed products.n1

**Scheme 4:**
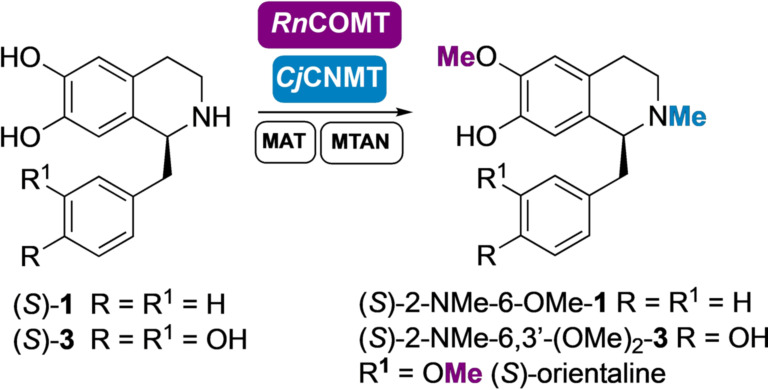
Synthesis of THIQs (*S*)‐2‐NMe‐6‐OMe‐**1** and (*S*)‐2‐NMe‐6,3′‐(OMe)_2_‐**3** on a laboratory preparative scale. (*S*)‐**1** and (S)‐**3** were formed in cascades starting from dopamine (see Schemes 1 and 3).

Finally, *N*‐methylation of (*S*)‐6‐OMe‐**1** and (*S*)‐6,3′‐(OMe)_2_‐**3** (*S*)‐nororientaline was also investigated for the total synthesis of (*S*)‐2‐NMe‐6‐OMe‐**1** and orientaline (*S*)‐2‐NMe‐6,3′‐(OMe)_2_‐**3** respectively (Scheme 4). *Cj*CNMT is known to methylate coclaurine and several natural THIQs, including norlaudanosoline.[^31^, ^40^, ^41^] In a first attempt to synthesize the desired products, the *N*‐methylation step was carried out in the same pot (in a cascade starting from dopamine), after completion of the *Rn*COMT step, by the addition of purified *Cj*CNMT (methylation steps shown in Scheme 4). (*S*)‐6,3′‐(OMe)_2_‐**3** was converted in 16 hours and (*S*)‐orientaline (*S*)‐2‐NMe‐6,3′‐(OMe)_2_‐**3** was isolated in 38 % yield (overall from dopamine). Compound (*S*)‐6‐OMe‐**1** was also accepted by *Cj*CNMT and converted into the corresponding *N*‐methylated product (*S*)‐2‐NMe‐6‐OMe‐**1** with a 46 % isolated yield (in a cascade starting from dopamine) and 66 % yield by HPLC analysis. Notably, this SAM‐dependent *N*‐MT was shown to be active if coupled with the SAM supply/SAH degradation system. To our delight, the *Cj*CNMT also showed comparable activity if added together with the *Rn*COMT in the same step, reducing reaction times; (*S*)‐2‐NMe‐6‐OMe)‐**1** and (*S*)‐orientaline (*S*)‐2‐NMe‐6,3′‐(OMe)_2_‐**3** were obtained in less than 48 hours in a two‐step one‐pot cascade from dopamine. In both cases traces (<5 %) of the minor isomers (*S*)‐2‐NMe‐7‐(OMe)‐**1** and (*S*)‐2‐NMe‐7,3′‐(OMe)_2_‐**3**, were noted.

Generally, *Rn*COMT is highly selective and the 6‐OH is preferentially methylated except in the case of spiro‐THIQs **9** and **10**, where a complete inversion of the selectivity was observed. To understand this pattern of regioselectivity, a selection of THIQs were docked into models of the *Rn*COMT and *Mx*SafC active sites using AutoDock Vina.^[42]^ The catechol‐binding pocket forms a deep groove with the magnesium ion proximal to highly conserved lysine residues (K144 and K145 in *Rn*COMT and *Mx*SafC respectively) involved in the deprotonation of the hydroxyl to be methylated. The catechol moiety coordinates with the magnesium ion, while the side chain establishes interactions in the outer region pointing either towards the so called “hydrophobic wall” or towards the solvent,[^35^, ^43^] defining the catechol position which is preferentially methylated. Nevertheless, none of our models could completely support the regioselectivity observed, presumably due to the high degree of reorientation of the active site around the specific substrate.^[30]^ However, some docking analysis with (*S*)‐6‐OMe‐**3** and *Rn*COMT (which exhibited high regioselectivity for the second methylation at 3′‐OH) and *Mx*SafC (which favored the second methylation at 4′‐OH) provided useful insights consistent with the experimental findings (SI Figure S6).

## Conclusion

In summary, we have developed in vitro enzymatic cascades for the synthesis of a wide range of methylated natural and non‐natural THIQ alkaloids, starting from commercially available and inexpensive building blocks. The application of an efficient SAM supply/SAH degradation system, together with the use of clarified lysates coupled with the NCS step, contribute to make the scale‐up of the whole process viable. Finally, methylated THIQ alkaloids in high enantiopurities such as (*S*)‐norreticuline, (*S*)‐coclaurine, (*S*)‐nororientaline, and (*S*)‐orientaline together with novel THIQs are readily available in high purity from a one‐pot cascade. The methylation step showed exceptional regioselectivity bearing in mind there is a degree of non‐enzymatic methylation (generally around 4 %) which has also been reported in the literature.[^30^, ^44^] An unprecedented dual activity has also been discovered for the methyltransferases *Rn*COMT and *Mx*SafC, which were found to be able to regioselectively methylate two different catechols in a single molecule. This unique activity permitted the regioselective synthesis of (*S*)‐nororientaline in one single step from the precursor (*S*)‐norlaudanosoline.

## Conflict of interest

The authors declare no conflict of interest.

## Supporting information

As a service to our authors and readers, this journal provides supporting information supplied by the authors. Such materials are peer reviewed and may be re‐organized for online delivery, but are not copy‐edited or typeset. Technical support issues arising from supporting information (other than missing files) should be addressed to the authors.

Supporting Information

## Footnotes

n1 Due to the difficulty of isolating each component and the complexity of the NMR data, we cannot completely rule out the presence of the regioisomer at the 3′‐OH position.

## References

[1] C. Straube , S. Derry , K. C. Jackson , P. J. Wiffen , R. F. Bell , S. Strassels , S. Straube , Cochrane Database Syst. Rev. 2014, 1–54. 10.1002/14651858.CD006601.pub4 PMC6513650 25234029

[2] H. Umihara , S. Yokoshima , M. Inoue , T. Fukuyama , Chem. Eur. J. 2017, 23, 6993–6995. 28378531 10.1002/chem.201701438

[3] M. A. Altinoz , G. Topcu , A. Hacimuftuoglu , A. Ozpinar , A. Ozpinar , E. Hacker , İ. Elmaci , Neurochem. Res. 2019, 44, 1796–1806. 31292803 10.1007/s11064-019-02837-x

[4] I. P. Singh , P. Shah , Expert Opin. Ther. Pat. 2017, 27, 17–36. 27623022 10.1080/13543776.2017.1236084

[5] J. D. Guzman , T. Pesnot , D. A. Barrera , H. M. Davies , E. McMahon , D. Evangelopoulos , P. N. Mortazavi , T. Munshi , A. Maitra , E. D. Lamming , R. Angell , M. C. Gershater , J. M. Redmond , D. Needham , J. M. Ward , L. E. Cuca , H. C. Hailes , S. Bhakta , J. Antimicrob. Chemother. 2015, 70, 1691–1703. 25656411 10.1093/jac/dkv010 PMC4498294

[6] M. Białoń , M. Żarnowska , L. Antkiewicz-Michaluk , A. Wąsik , Psychopharmacology (Berl.) 2020, 237, 1577–1593. 32076746 10.1007/s00213-020-05484-1 PMC7239818

[7] T. Xu , T. Kuang , H. Du , Q. Li , T. Feng , Y. Zhang , G. Fan , Pharmacol. Res. 2020, 152, 104632. 31911246 10.1016/j.phrs.2020.104632

[8] L. Braconi , G. Bartolucci , M. Contino , N. Chiaramonte , R. Giampietro , D. Manetti , M. G. Perrone , M. N. Romanelli , N. A. Colabufo , C. Riganti , S. Dei , E. Teodori , J. Enzyme Inhib. Med. Chem. 2020, 35, 974–992. 32253945 10.1080/14756366.2020.1747449 PMC7178819

[9] F. Ghirga , M. Mori , A. Bonamore , L. Calisti , A. Boffi , A. Macone , I. D′Acquarica , B. Botta , Int. J. Mol. Sci. 2017, 18, 2464. 29156609 10.3390/ijms18112464 PMC5713430

[10] B. R. Lichman , M. C. Gershater , E. D. Lamming , T. Pesnot , A. Sula , N. H. Keep , H. C. Hailes , J. M. Ward , FEBS J. 2015, 282, 1137–1151. 25620686 10.1111/febs.13208 PMC4413047

[11] B. R. Lichman , J. Zhao , H. C. Hailes , J. M. Ward , Nat. Commun. 2017, 8, 14883. 28368003 10.1038/ncomms14883 PMC5382262

[12] R. Roddan , G. Gygli , A. Sula , D. Méndez-Sánchez , J. Pleiss , J. M. Ward , N. H. Keep , H. C. Hailes , ACS Catal. 2019, 9, 9640–9649.

[13] B. M. Ruff , S. Bräse , S. E. O'Connor , Tetrahedron Lett. 2012, 53, 1071–1074. 22966211 10.1016/j.tetlet.2011.12.089 PMC3435518

[14] J. J. Maresh , S. O. Crowe , A. A. Ralko , M. D. Aparece , C. M. Murphy , M. Krzeszowiec , M. W. Mullowney , Tetrahedron Lett. 2014, 55, 5047–5051.

[15] M. Nishihachijo , Y. Hirai , S. Kawano , A. Nishiyama , H. Minami , T. Katayama , Y. Yasohara , F. Sato , H. Kumagai , Biosci. Biotechnol. Biochem. 2014, 78, 701–707. 25036970 10.1080/09168451.2014.890039

[16] J. Zhao , B. R. Lichman , J. M. Ward , H. C. Hailes , Chem. Commun. 2018, 54, 1323–1326. 10.1039/c7cc08024g PMC5804477 29345260

[17] Y. Wang , N. Tappertzhofen , D. Méndez-Sánchez , M. Bawn , B. Lyu , J. M. Ward , H. C. Hailes , Angew. Chem. Int. Ed. 2019, 58, 10120–10125; 10.1002/anie.201902761 31100182

[18] T. Gurkok , E. Ozhuner , I. Parmaksiz , S. Özcan , M. Turktas , A. İpek , I. Demirtas , S. Okay , T. Unver , Front. Plant Sci. 2016, 7, 1–11. 26909086 10.3389/fpls.2016.00098 PMC4754624

[19] G. Lunn , B. J. Banks , R. Crook , N. Feeder , A. Pettman , Y. Sabnis , Bioorg. Med. Chem. Lett. 2011, 21, 4608–4611. 21737265 10.1016/j.bmcl.2011.05.132

[20] M. Egbertson , G. B. McGaughey , S. M. Pitzenberger , S. R. Stauffer , C. A. Coburn , S. J. Stachel , W. Yang , J. C. Barrow , L. A. Neilson , M. McWherter , D. Perlow , B. Fahr , S. Munshi , T. J. Allison , K. Holloway , H. G. Selnick , Z. Yang , J. Swestock , A. J. Simon , S. Sankaranarayanan , D. Colussi , K. Tugusheva , M. T. Lai , B. Pietrak , S. Haugabook , L. Jin , I. W. Chen , M. Holahan , M. Stranieri-Michener , J. J. Cook , J. Vacca , S. L. Graham , Bioorg. Med. Chem. Lett. 2015, 25, 4812–4819. 26195137 10.1016/j.bmcl.2015.06.082

[21] M. A. Walker , Bioorg. Med. Chem. Lett. 2017, 27, 5100–5108. 29100802 10.1016/j.bmcl.2017.09.041

[22] J. Claesen , M. Bibb , Proc. Natl. Acad. Sci. USA 2010, 107, 16297–16302. 20805503 10.1073/pnas.1008608107 PMC2941285

[23] J. Lee , Y. Hao , P. M. Blair , J. O. Melby , V. Agarwal , B. J. Burkhart , S. K. Nair , D. A. Mitchell , Proc. Natl. Acad. Sci. USA 2013, 110, 12954–12959. 23878226 10.1073/pnas.1306101110 PMC3740862

[24] L. Najmanová , E. Kutejová , J. Kadlec , M. Polan , J. Olšovská , O. Benada , J. Novotná , Z. Kameník , P. Halada , J. Bauer , J. Janata , ChemBioChem 2013, 14, 2259–2262. 24166757 10.1002/cbic.201300389

[25] F. Sato , T. Tsujita , Y. Katagiri , S. Yoshida , Y. Yamada , Eur. J. Biochem. 1994, 225, 125–131. 7925429 10.1111/j.1432-1033.1994.00125.x

[26] T. Frenzel , M. H. Zenk , Phytochemistry 1990, 29, 3505–3511.

[27] T. Pesnot , M. C. Gershater , J. M. Ward , H. C. Hailes , Chem. Commun. 2011, 47, 3242–3244. 10.1039/c0cc05282e 21270984

[28] J. Vidgren , L. A. Svensson , A. Liljas , Nature 1994, 368, 354–358. 8127373 10.1038/368354a0

[29] T. Morishige , T. Tsujita , Y. Yamada , F. Sato , J. Biol. Chem. 2000, 275, 23398–23405. 10811648 10.1074/jbc.M002439200

[30] J. Siegrist , J. Netzer , S. Mordhorst , L. Karst , S. Gerhardt , O. Einsle , M. Richter , J. N. Andexer , FEBS Lett. 2017, 591, 312–321. 27990630 10.1002/1873-3468.12530

[31] M. R. Bennett , M. L. Thompson , S. A. Shepherd , M. S. Dunstan , A. J. Herbert , D. R. M. Smith , V. A. Cronin , B. R. K. Menon , C. Levy , J. Micklefield , Angew. Chem. Int. Ed. 2018, 57, 10600–10604; 10.1002/anie.201805060 PMC6099451 29791083

[32] S. Mordhorst , J. Siegrist , M. Müller , M. Richter , J. N. Andexer , Angew. Chem. Int. Ed. 2017, 56, 4037–4041; 10.1002/anie.201611038 28170142

[33] J. Siegrist , S. Aschwanden , S. Mordhorst , L. Thöny-Meyer , M. Richter , J. N. Andexer , ChemBioChem 2015, 16, 2576–2579. 26437744 10.1002/cbic.201500410

[34] U. Kaulmann , K. Smithies , M. E. B. Smith , H. C. Hailes , J. M. Ward , Enzyme Microb. Technol. 2007, 41, 628–637.

[35] M. J. Vanden Eynden , K. Kunchithapatham , J. P. Stambuli , J. Org. Chem. 2010, 75, 8542–8549. 21090691 10.1021/jo1019283

[36] D. R. Thakker , C. Boehlerts , K. L. Kirks , R. Antkowiak , C. R. Creveling , J. Biol. Chem. 1986, 261, 178–184. 3753600

[37] B. R. Lichman , E. D. Lamming , T. Pesnot , J. M. Smith , H. C. Hailes , J. M. Ward , Green Chem. 2015, 17, 852–855.

[38] A. J. Vlietinck , T. De Bruyne , S. Apers , L. A. Pieters , Planta Med. 1998, 64, 97–109. 9525100 10.1055/s-2006-957384

[39] A. Ruiz-Olalla , M. A. Würdemann , M. J. Wanner , S. Ingemann , J. H. Van Maarseveen , H. Hiemstra , J. Org. Chem. 2015, 80, 5125–5132. 25909585 10.1021/acs.joc.5b00509

[40] K. B. Choi , T. Morishige , F. Sato , Phytochemistry 2001, 56, 649–655. 11314949 10.1016/s0031-9422(00)00481-7

[41] K. B. Choi , T. Morishige , N. Shitan , K. Yazaki , F. Sato , J. Biol. Chem. 2002, 277, 830–835. 11682473 10.1074/jbc.M106405200

[42] O. Trott , A. J. Olson , J. Comput. Chem. 2010, 31, 455–461. 19499576 10.1002/jcc.21334 PMC3041641

[43] B. J. C. Law , M. R. Bennett , M. L. Thompson , C. Levy , S. A. Shepherd , D. Leys , J. Micklefield , Angew. Chem. Int. Ed. 2016, 55, 2683–2687; 10.1002/anie.201508287 PMC4770447 26797714

[44] B. Rydberg , T. Lindahl , EMBO J. 1982, 1, 211–216. 7188181 10.1002/j.1460-2075.1982.tb01149.x PMC553022

